# Supplementary figures and images for: Traffic Instabilities in Self-Organized Pedestrian Crowds
Source: PLoS Comput Biol. 2012 Mar 22;8(3):e1002442. doi: 10.1371/journal.pcbi.1002442 (PMC3310728; doi:10.1371/journal.pcbi.1002442)

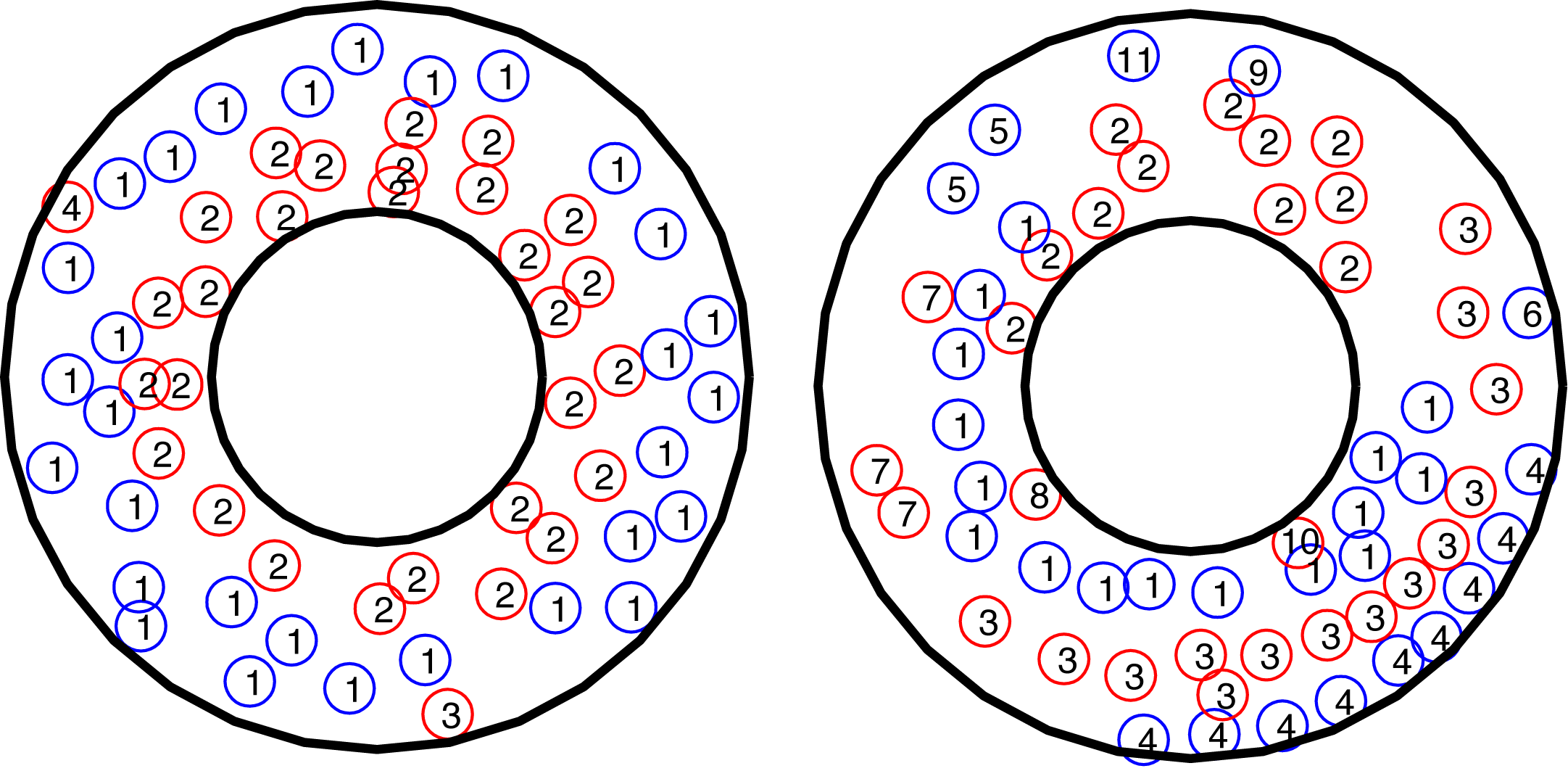

Supplement: Figure S1 — Illustration of the outcome of the clustering technique for a replication with N = 60 pedestrians, where a cluster number has been automatically attributed to each individual. A well-organized situation is shown on the left (4 clusters), and a disorganized state is shown on the right (11 clusters). Blue pedestrians turn clockwise and red pedestrians anti-clockwise. (TIF) [file pcbi.1002442.s001.tif]

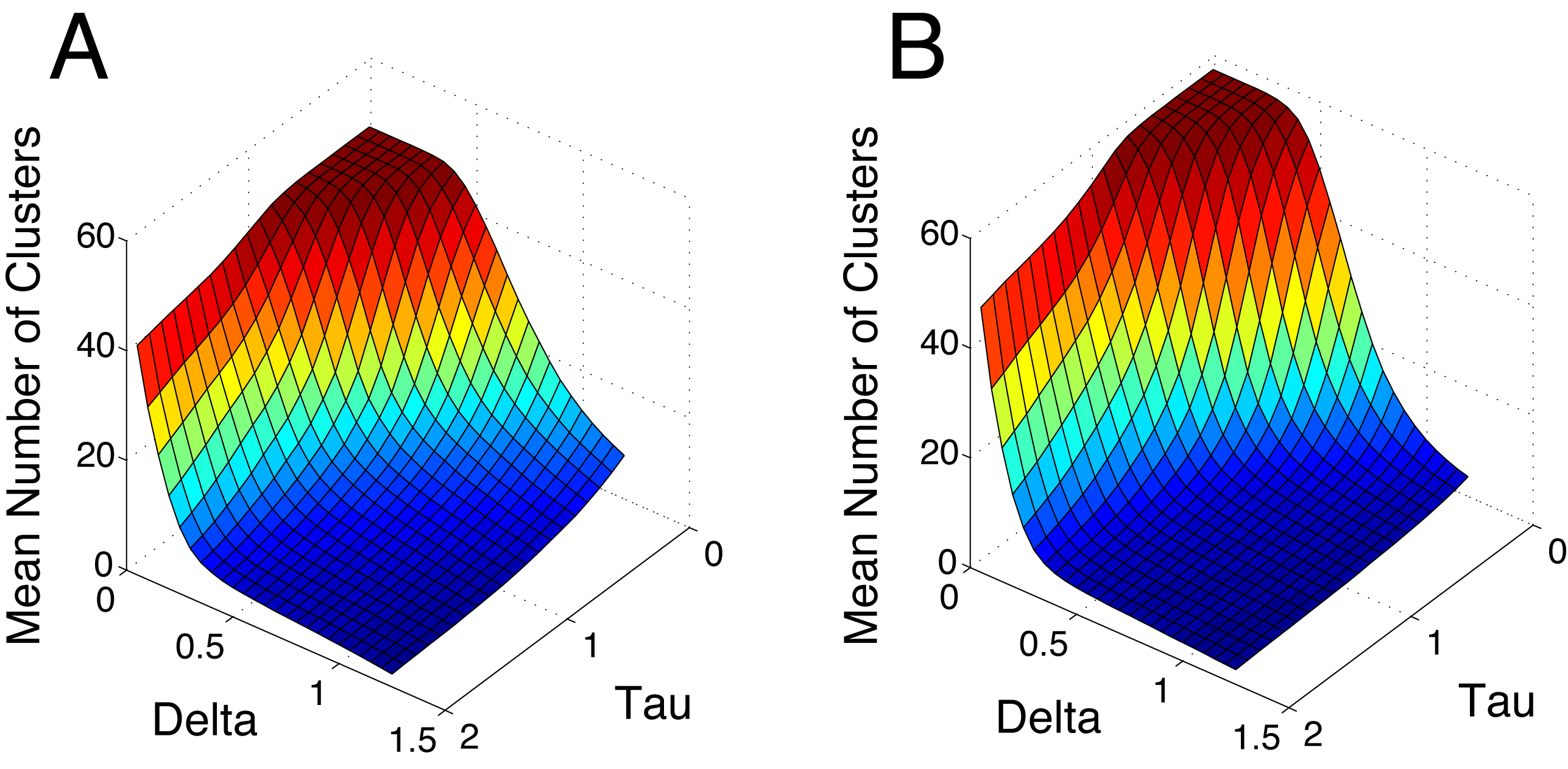

Supplement: Figure S2 — Surface plot of the mean number of clusters detected for 50 pedestrians experiments (A), and 60 pedestrians experiments (B). (TIF) [file pcbi.1002442.s002.tif]
