# Supplementary material for: Traffic Instabilities in Self-Organized Pedestrian Crowds
Source: PLoS Comput Biol. 2012 Mar 22;8(3):e1002442. doi: 10.1371/journal.pcbi.1002442 (PMC3310728; doi:10.1371/journal.pcbi.1002442)
Supplement: Text S1 — Parametric sensitivity study for the clustering method. (DOC) [file pcbi.1002442.s003.doc]

## Parametric sensitivity study for the clustering method

**Parameter Range and Indicator**

The values chosen for our parameters, $d$ and $t$, have been set mainly based on biological considerations. However a parametric sensitivity study is crucial to gather information about eventual ranges of parameter values of lesser sensitivity. The study was performed for (in seconds) and (in meters). The number of clusters was used as an indicator. In order to compare one value for each $\left\{ d,t \right\}$ couple, we take a mean of the indicator over the ”meaningful” time of all experimental recordings with the same number of pedestrians. That is, from 5 seconds after the pedestrians start walking to 50 seconds later. We write $N_{L}^{K}(t,d)$ the mean number of clusters for the ensemble of all *K-*pedestrians experiments. **Figure S4** shows the surfaces drawn by $N_{L}^{50}(t,d)$ and $N_{L}^{60}(t,d)$.

**Lesser Sensitivity Window**

We observe a flat region in the $t,d$ plane. This result suggests that any set of parameters chosen in this area will yield a similar N_L_ function. However, more than minimizing parameter sensitivity, we need our detection of clusters to make sense. That is why we define a lesser (but not least) sensitivity window that conciliates a low sensitivity to $t$ and $d$, and a somewhat realistic range of values for these same parameters. This is the following window:

Within this window, the relative variation of $N_{L}^{60}$ with both $t$ and $d$ does not exceed 7% and that of $N_{L}^{50}$ do not exceed 10%.
